# Network Pharmacology and Absolute Bacterial Quantification-Combined Approach to Explore the Mechanism of Tianqi Pingchan Granule Against 6-OHDA-Induced Parkinson’s Disease in Rats

**DOI:** 10.3389/fnut.2022.836500

**Published:** 2022-05-06

**Authors:** Zhihua Liu, Jiahao Zhao, Shuyuan Yang, Yu Zhang, Lu Song, Na Wu, Zhenguo Liu

**Affiliations:** Department of Neurology, Xinhua Hospital Affiliated to Shanghai Jiao Tong University School of Medicine, Shanghai, China

**Keywords:** gut microbiota, microbiota-gut-brain axis, network pharmacology, traditional Chinese medicine, Parkinson’s disease

## Abstract

Parkinson’s disease (PD) is the second most common neurodegenerative disease. Tianqi Pingchan Granule (TPG) is a clinically effective formula of traditional Chinese medicine to treat PD. However, the therapeutic effect and underlying mechanisms of TPG in PD remain unclear. Based on network pharmacology, the corresponding targets of TPG were identified using the Traditional Chinese Medicine Database and Analysis Platform Database. Differentially expressed genes in PD were obtained from the Therapeutic Target Database, Online Mendelian Inheritance in Man, GeneCards, and DrugBank databases. The protein-protein interaction (PPI) networks of intersected targets were constructed using the STRING database and visualized using Cytoscape. Gene Ontology (GO) and Kyoto Encyclopedia of Genes and Genomes (KEGG) pathway enrichment analyses were performed, and the pathways directly related to the pathogenesis of PD were integrated manually. Furthermore, *in vivo* studies were carried out based on network pharmacology. The gut microbiota, peripheral inflammatory cytokines, and glia-mediated neuroinflammation in substantia nigra were evaluated. A total of 99 target genes were intersected between targets of TPG and deferentially expressed genes in PD. The PPI network analysis indicated the proinflammatory cytokine as essential targets. GO and KEGG analyses indicated that inflammatory response and its related signaling pathways were closely associated with TPG-mediated PD treatment. *In vivo* studies revealed that class Negativicutes and order Selenomonadales decreased, whereas class Mollicutes, order Enterobacteriales, and Mycoplasmatales increased in fecal samples of PD rats *via* 16S rRNA sequence analysis. Furthermore, the function prediction methods purposely revealed that TPG therapy may be involved in flavonoid biosynthesis, which have anti-inflammatory properties. In addition, *in vivo* studies revealed that TPG exposure was found to not only attenuate the production of peripheral inflammatory cytokines but also inhibit the activation of microglia and astrocytes in substantia nigra of PD rats. Through network pharmacology and *in vivo* experiment-combined approach, the mechanisms of TPG in the treatment of PD were revealed, and the role of TPG in the regulation of gut microbiota and inflammatory response was confirmed.

## Introduction

Parkinson’s disease (PD) is one of the most common neurodegenerative disorders commonly seen among the elderly (1). PD is characterized by both motor and non-motor symptoms, including bradykinesia, resting tremor, rigidity, and gastrointestinal dysfunctions, such as constipation (2).

Current therapies for PD selectively modulate established targets, such as levodopa and dopamine agonists, have declined efficacy after long-term use (3). The old concept of developing selective agents for a single target does not fit with the medical need of most neurological diseases (4). Thus, there exists an urgent unmet need to develop multitarget drugs that have advantages of higher efficacy, improved safety profile, and simpler administration. As conventionally medical therapy is of limited relief and potential side effects, traditional Chinese medicine (TCM) has attracted growing public and professional attention in clinical PD management (5) due to the advantages of multicomponent, multipathway, and multitarget synergies.

Tianqi Pingchan Granule (TPG) is a clinically effective formula of TCM that has been used in our hospital for the management of PD. TPG is modified based on classic TCM prescriptions “Mi Fang Ding Zhen Wan” compiled by Kentang Wang of the Ming Dynasty (6), which is a safe, reliable, and effective method to alleviate motor symptoms of PD. Early clinical studies in our hospital showed that TPG has a significant therapeutic effect on constipation and motor complication of patients with PD, such as L-dopa-induced dyskinesia, and is safe for consumption. However, the underlying molecular mechanisms of TPG in treating PD remain unclear and warrant further investigation. However, the precise protective role and mechanism of TPG against PD remain unclear.

**FIGURE 1:**
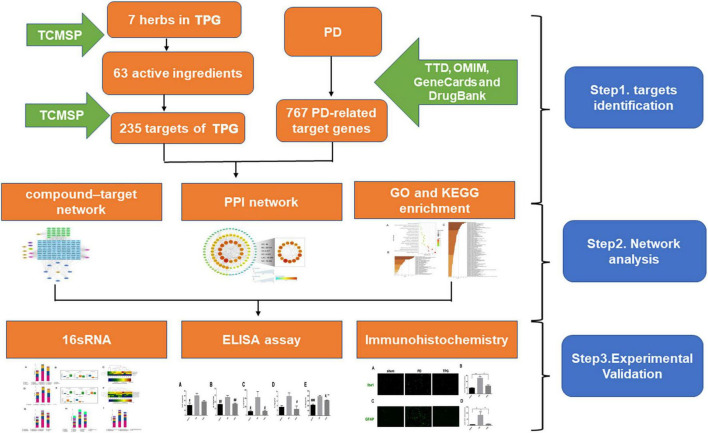
The technical strategy of this study.

Network pharmacology offers an ideal opportunity to understand the complex multiple-component drug system of TCM and the mechanisms through which TCM functions *in vivo* (7). Therefore, in this study a network pharmacological approach was used to investigate the pharmacological network of TPG on PD to predict the active compounds and potential targets and pathways. In addition, *in vivo* experiments were also conducted to validate the potential underlying mechanism of TPG on PD as predicted by the network pharmacology approach. The detailed technical strategy of this study is shown in Figure 1.

## Materials and Methods

### Network Pharmacology Analysis of Tianqi Pingchan Granule

#### Compound Profiling and Disease Target Identification

The pharmacological information of TPG was retrieved from the Traditional Chinese Medicine Database and Analysis Platform (TCMSP)^1^ (8). The components of each herb with a drug-likeness (DL) ≥ 0.18 and an oral bioavailability (OB) ≥ 30% (9, 10) were selected as bioactive ingredients while their target genes, as cataloged in the databases, were identified as TPG targets. Then, the target corresponding to the compounds screened from the TCMSP database was standardized in UniProt.^2^ Furthermore, to identify targets of TPGs, a compound–target network was established *via* Cytoscape version (ver.) 3.8.0 (11).

Disease-related genes were obtained from the Therapeutic Target Database (TTD)^3^ (12), Online Mendelian Inheritance in Man (OMIM)^4^ (13), GeneCards^5^ (14), and DrugBank^6^ (15) and combined into a list of PD-related genes.

#### Network Establishment

Subsequently, the effect of TPG on PD was further analyzed by making Venn maps of 235 TPG predicted targets and 767 PD-related genes obtained using the above methods. The intersection was determined using Venny ver. 2.1^7^ to obtain the therapeutic targets of TPG for PD. The data were then exported to the Cytoscape ver. 3.8.0 to construct a ‘‘Drug-Target-Disease’’ network. The STRING ver. 11.0b database^8^ was used to generate a protein–protein interaction (PPI) network (16). We selected PPI data for “*Homo sapiens*” with a confidence score ≥0.4 to construct the PPI network, and Textmining, Experiments, Databases, Co-expression, Neighborhood, Gene Fusion, and Co-occurrence were selected to analyze the PD -related targets in TPG interaction. The network was treated as undirected to identify the core candidate targets of TPG against PD. We set the topological parameter medians of degree (DC), betweenness (BC), closeness (CC), eigenvector (EC), local average connectivity-based method (LAC), and network (NC) as screen parameters to construct core PPI network.

#### Pathway and Functional Enrichment Analysis

Gene Ontology (GO) and Kyoto Encyclopedia of Genes and Genomes (KEGG) enrichment analyses of TPG’s core potential therapeutic targets were performed using metascape.^9^ The bubble chart was plotted with R ver. 4.0.3 (R Foundation for Statistical Computing, Vienna, Austria)^10^ and the ggplot2 package.

### Experimental Validation

#### Materials and Reagents

Tianqi Pingchan Granule is one of the TCM composed of six kinds of plant-derived medicines, such as *Astragalus mongholicus* Bunge [Fabaceae] (HQ), *Rehmannia glutinosa* (Gaertn.) DC. [Orobanchaceae] (SD), *Paeonia lactiflora* Pall. [Paeoniaceae] (BS), *Angelica sinensis (*Oliv.) Diels [Apiaceae] (DG), *Uncaria rhynchophylla* (Miq.) Miq. [Rubiaceae] (GT), and *Gastrodia elata* Blume [Orchidaceae] (TM), and one kind of animal-derived medicine, such as *Bombyx batryticatus* [Bombycidae] (JC), the dried larva of *Bombyx mori* L. (4th–5th instars) infected with *Beauveria bassiana* Vuill. These seven medicines were used to synthesize TPG in Sichuan New GreenMedicine Science and Technology Development Co., Ltd., (Chendu, China) under the guidance of the Chinese Pharmacopeia 2020 edition. HQ was picked from China’s Gansu province in spring and autumn. SD was picked from China’s Henan province in autumn. BS was picked from China’s Anhui province in summer and autumn. DG was picked from China’s Gansu province in late autumn. GT was picked from China’s Jiangxi province in spring and autumn. TM was picked from China’s Anhui province during the period from the beginning of winter to the next year before Qingming Festival. JC was collected from China’s Zhejiang province in autumn and winter. TPG was dissolved in pure water and injected into intragastric. The doses of TPG in our animal studies were selected based on our previous clinical study, and dose ranges were therapeutically relevant.

6-Hydroxydopamine (6-OHDA), apomorphine, L-dopa, and benserazide were obtained from Sigma Chemical Co., (St. Louis, MO, United States).

#### Animals and Establishment of Parkinson’s Disease Model

Adult male Sprague-Dawley rats weighing 200–250 g were used, and all rats were kept under standard laboratory conditions as previously described (17). The protocols of this study were reviewed and approved by the Ethical Committee of the Medical School of Shanghai Jiao Tong University (the ethical clearance number: XHEC-NSFC-2019-210). The methods were carried out in compliance with the approved guidelines and regulations of the National Institutes of Health for the care and use of laboratory animals.

To establish a rat model of PD, 6-OHDA was injected into the right middle forebrain bundle of each rat. Animal surgical procedures were performed as previously reported (17). Three weeks after the 6-OHDA injection, rats with a rotating frequency >7 turns/min in apomorphine-induced rotation tests were selected as successful PD rat models.

#### Animal Experimental Design and Drug Treatment

All rats were randomly divided into three groups (*n* = 4 rats per group): sham, PD, and TPG. TPG group rats were gavaged with TPG (5.6 g/kg) once daily at the same time. The control group of sham-lesioned rats and PD group rats was gavaged with sterile saline alone. The dose of TPG was based on our previous clinical study (18).

#### Fecal Microbiota: Absolute Quantification of 16S rRNA

For collecting feces, all rats were placed individually in empty autoclaved cages and allowed to defecate freely in the morning after the day of last treatment. Once feces were formed of each mouse, they were collected immediately in individual sterile EP tubes on ice and then stored at −80°C until next usage.

Absolute quantification of 16S rRNA amplicon sequencing was performed by Genesky Biotechnologies Inc., Shanghai, (China). Total genomic DNA was extracted using the FastDNA^®^ SPIN Kit for Soil (MP Biomedicals, Santa Ana, CA, United States) according to the instructions of the manufacturer. The integrity of genomic DNA was detected through agarose gel electrophoresis, and the concentration and purity of genomic DNA were detected through the Nanodrop 2000 and Qubit3.0 Spectrophotometer. Multiple spike-ins with identical conserved regions to natural 16S rRNA genes, and variable regions replaced by random sequences with ∼40% GC content were artificially synthesized. Then, an appropriate proportion of spike-ins mixture with known gradient copy numbers were added to the sample DNA. The V3-V4 hypervariable regions of the 16S rRNA gene and spike-ins were amplified with the primers 341F (5′-CCTACGGGNGGCWGCAG-3′) and 805R (5′-GACTACHVGGGTATCTAATCC-3′), and then sequenced using Illumina NovaSeq 6000 sequencer.

The raw read sequences were processed in QIIME2 (19). The adaptor and primer sequences were trimmed using the cutadapt plugin. DADA2 plugin was used for quality control and to identify amplicon sequence variants (ASVs) (20). Taxonomic assignments of ASV representative sequences were performed with confidence threshold of 0.8 by a pretrained Naive Bayes Classifier, which was trained on the Greengenes ver. 13.8. Then, the spike-in sequences were identified and reads were counted. The standard curve for each sample was generated based on the read counts vs. spike-in copy number, and the absolute copy number of each ASV in each sample was calculated by using the read counts of the corresponding ASV. As the spike-in sequence is not a component of the sample flora, the spike-in sequence needs to be removed in the subsequent analysis (21).

Functional prediction analysis was assessed using PICRUSt2.

### ELISA Assay

ELISA was utilized to analyze the concentrations of the serum inflammatory cytokines according to instructions of the manufacturer. The whole blood was centrifuged at 2,000 × *g* for 20 min at 4°C, and the supernatant was collected for the further measurement of interleukin (IL)-1, IL-2, IL-4, IL-6, and tumor necrosis factor-ɑ (TNF-ɑ) using ELISA kits (Shanghai Westang Biotechnology, Shanghai, China).

### Immunohistochemistry

Immunohistochemistry (IHC) was performed as previously described (17). Rat brains were fixed by 4% cold paraformaldehyde and cryoprotected in 30% sucrose in phosphate-buffered saline. Coronal sections (cut thickness: 3 μm) were cut on a freezing microtome, then immunoreacted with primary antibodies: anti-ionized calcium-binding adaptor molecule 1 (Iba1) (Servicebio, GB13105-1, Wuhan, China, 1:300) and anti-glial fibrillary acidic protein (GFAP, Servicebio, Wuhan, China, GB11096, 1:1,000) for immunolabeling. The sections were visualized using a fluorescence microscope (Nikon Eclipse C1, Japan) to examine the extent of microgliosis and astrogliosis, and representative photomicrographs of ipsilateral striatum regions were taken under a 40 × magnification objective. The mean fluorescence intensity of GFAP was used to evaluate the extent of microgliosis and astrogliosis and measured with the ImageJ software (National Institutes of Health, Bethesda, MD, United States).

### Statistical Analysis

Data were analyzed using GraphPad Prism ver. 6.0 software. Data of the Western blot, IHC data, and ELISA are presented as mean ± SEM and analyzed by one-way ANOVA followed by Sidak’s multiple-comparisons test. Statistical significance was set at *p* < 0.05.

The sample size determination in the animal study was calculated based on our preexperiment data and the power test for sample size analysis by using PASS ver. 15.0 software. The power for the primary endpoint serum concentrations of inflammatory cytokines was calculated based on a one-way ANOVA test with a significance level of 5%. With an average sample size of 4 subjects and a total sample size of 12 subjects, the animal study will have more than 90% power to detect a difference among three groups.

## Results

### Active Ingredients of Tianqi Pingchan Granule

In our initial search of the TCMSP database, we identified 73 active ingredients of potential relevance (Supplementary Table 1). Of these, we selected 20 ingredients in HQ, 2 ingredients in SD 13 ingredients in BS, 2 ingredients in DG, 33 ingredients in GT, 1 ingredient in JC, and 2 ingredients in TM based upon their OB and DL values. Of note, there were six common components shared in these seven herbs, namely, Mairin, 1,7-dihydroxy-3,9-dimethoxy pterocarpene, quercetin, sitosterol, stigmasterol, and beta-sitosterol. After eliminating the overlaps, 63 components were chosen as candidate bioactive components for further analyses and the detailed information is shown in Supplementary Table 2.

### Identification of Tianqi Pingchan Granule and Parkinson’s Disease Targets

The gene targets of the 63 active compounds in TPG were retrieved from the TCMSP database, and 1,542 targets were retrieved. After eliminating the overlaps, we identified 235 TPG targets, which included TNF, IL-1β, IL-2, IL-4, and IL-6. The detailed information is shown in Supplementary Table 3. In addition, after eliminating the overlaps, the TTD, OMIM, GeneCards, and DrugBank databases were used to identify 767 PD-related target genes (Supplementary Table 4).

### Network Analysis of Targets

**FIGURE 2:**
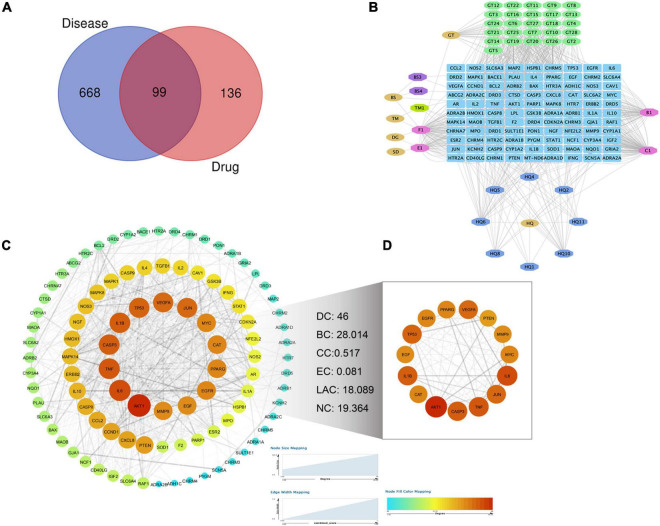
Screening of targets for Parkinson’s disease treated by TPG. **(A)** Venn diagram of drug targets and disease targets. **(B)** The compound-target network for TPG on Parkinson’s disease. Blue indicates drug-disease intersection targets, other colors indicate active compounds of TPG, and pink indicates overlapped active compounds of TPG. **(C)** PPI network of Parkinson’s disease-related targets in TPG. **(D)** Core PPI network of Parkinson’s disease-related targets in TPG extracted from panel **(C)**. The size and color of nodes represent their degree values, which change from blue to orange representing values from low to high. The width of edges represents combined scores between the nodes. DC, degree centrality; BC, betweenness centrality; CC, closeness centrality; EC, eigenvector centrality; LAC, local average connectivity-based method; NC, network centrality.

As shown in Figure 2A, the closed-loop form of the fixed position was used to represent all the drug target genes and disease-related genes. A total of 99 interaction targets were obtained in the Venn diagram. A “TPG-Targets-PD” network was constructed using Cytoscape (Figure 2B). The top five compounds with the highest degree centrality (DC) were quercetin, beta-sitosterol, kaempferol, stigmasterol, and 7-*O*-methylisomucronulatol. To explore the interactions among the above targets in-depth, we constructed a PPI network. The primary network had 98 nodes and 1,275 edges (Figure 2C). Next, the CytoNCA plug-in was used to calculate topological parameters and extract the core PPI network. The core PPI network consists of 15 nodes and 105 edges (Figure 2D). TNF, IL-1β, and IL-6 were identified in the core network, suggesting that these targets were worthy of further investigation in the context of PD treatment using TPG.

### Predicting Functional Enrichment Analysis for Tianqi Pingchan Granule

**FIGURE 3:**
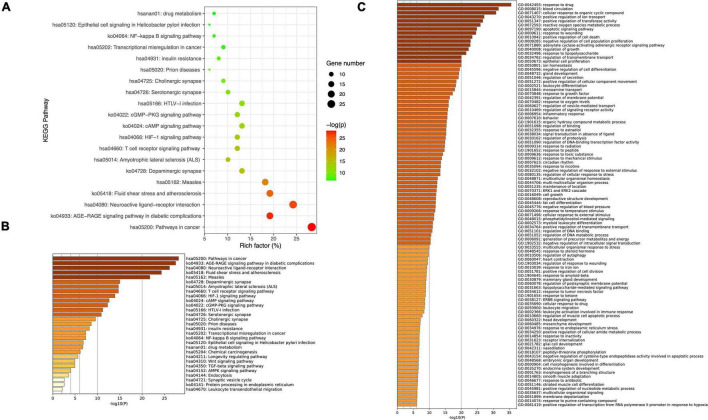
Kyoto Encyclopedia of Genes and Genomes pathway and GO enrichment of core candidate targets of TPG against Parkinson’s disease. **(A)** Top 20 KEGG pathway enrichment. **(B)** Heatmaps of KEGG pathway enrichment of core candidate targets of TPG against Parkinson’s disease. **(C)** Heatmaps of GO_BP enrichment.

Metascape was used to carry out functional enrichments of GO and KEGG pathways of the 99 overlapping TPG and PD targets (Figure 3). The top 20 significantly enriched pathways of TPG on PD are shown in Figure 3A. KEGG analysis revealed that many target genes were strongly associated with the NF-κB signaling pathway and T cell receptor signaling pathway (Figures 3A,B). By observing the results of Biological Process terms of GO analysis (Figure 3C), interaction targets were mainly associated with response to lipopolysaccharide, regulation of secretion, the inflammatory response, lipopolysaccharide-mediated signaling pathway, response to TNF, and glial cell development.

### Experimental Validation of Network Pharmacology

Gut microbiota dysbiosis can play a critical role in propagating peripheral inflammatory response and exacerbate the inflammatory environment in the brain through the microbiota-gut-brain axis (22, 23). Thus, to validate network pharmacology, the gut microbiota, peripheral inflammatory cytokines, and glia-mediated neuroinflammation in the lesioned substantia nigra of PD rats were evaluated.

### Tianqi Pingchan Granule-Induced Changes in Gut Microbiota

**FIGURE 4:**
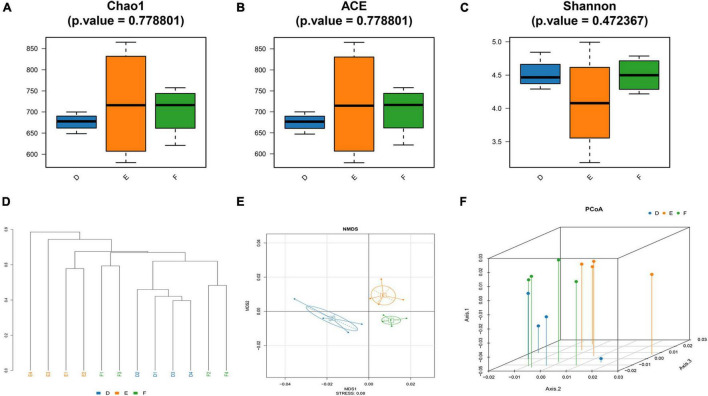
Changes in the diversity of the gut microbiota (*n* = 4/group): **(A–C)** the Chao1 index, ACE index, and Shannon index of three groups; **(D)** Samples Cluster Tree analysis based on unweighted pair group method with arithmetic mean method; **(E)** non-metric multidimensional scaling method (NMDS) is to simplify the research objects (samples or variables) in multidimensional space to low-dimensional space for positioning, analysis, and classification. **(F)** Principal coordinates analysis (PCoA).

The rarefaction curve is used to reflect the rationality of sequencing data and indirectly the species richness. As shown in Supplementary Figure 1, the rarefaction curve for each group indicated that the amount of sequencing data is sufficient. The alpha diversity indices of Chao1 and ACE, representative of microbial richness, tended to be lower in the sham group (D) rats and TPG group (F) rats compared to the PD group (E) rats (Figures 4A,B), but there were no significant differences between the three groups, suggesting that the number of samples needs to be expanded. Similarly, other alpha diversity indices of Shannon, representative of microbial diversity, tended to be higher in the sham group (D) rats and TPG group (F) rats compared to the PD group (E) rats (Figure 4C), but there were no significant differences between the three groups.

**FIGURE 5:**
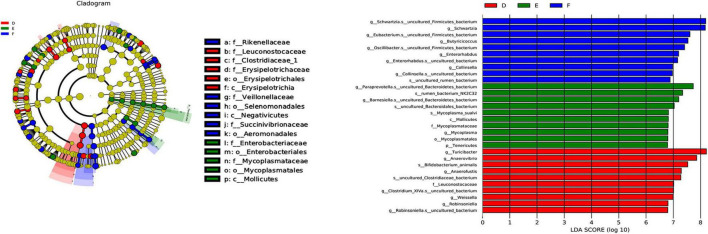
Major differential microbial species (*n* = 4/group): Taxonomic cladogram obtained from LEfSe of six groups. Biomarker taxa are highlighted with colored circles and shaded areas. Each circle’s diameter reflects the abundance of those taxa in the community.

To further analyze the similarity or difference of the composition of the gut microbiota of the three groups, Samples Cluster Tree analysis was performed. Strikingly, it can be clearly seen from Figure 4D that the samples were clustered by subject, indicating that microbial composition of the PD group was different from that of the sham group and TPG group. Also, other measures of beta diversity indices, including non-metric multidimensional scaling method (NMDS) and principal coordinates analysis (PCoA) (Figures 4E,F), were similar to Samples Cluster Tree analysis. It can be found that the differences between the TPG group and sham group were not obvious, indicating that the gut microbiota composition of the TPG control and the sham group was similar. In addition, the linear discriminant analysis effect size (LEfSe) was used to identify the specific phylotypes responding to the sham, PD, and TPG groups. The differences in the dominant members of the microbiota between the PD group and other groups are shown in Figure 5.

**FIGURE 6:**
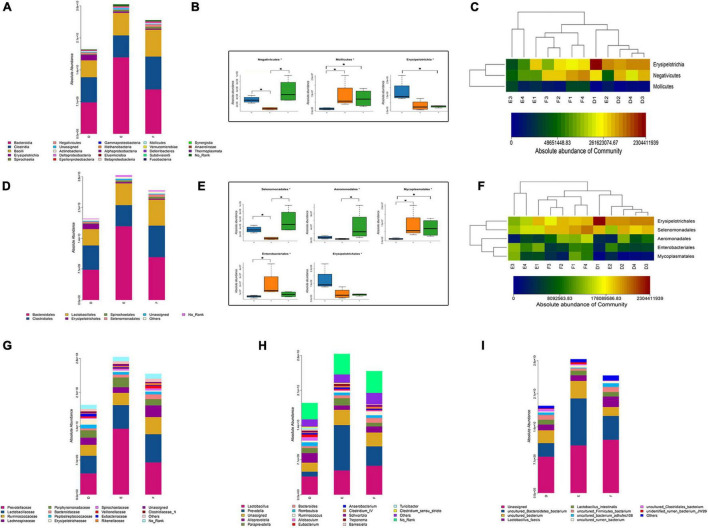
Composition of the gut microbiota (*n* = 4/group): **(A)** the absolute abundance of major bacterial taxa at the class level; **(B,C)** significant different bacteria at the class level. The data were analyzed by one-way ANOVA (*p < 0.05); **(D)** the absolute abundance of major bacterial taxa at the order level; **(B,C)** significant different bacteria at the order level. The data were analyzed by Kruskal–Wallis (*p < 0.05); **(G–I)** the absolute abundance of major bacterial taxa at the family, genus, and species levels.

From the class-level analysis (Figures 6A–C), a significant decrease was observed in the abundance of class Negativicutes, while class Mollicutes were increased in the PD group rats compared to the sham group rats. Interestingly, there were obvious increases in Negativicutes in the TPG group rats than in the PD group rats. At the order level (Figures 6D–F), decreased Selenomonadales and increased Enterobacteriales and Mycoplasmatales were observed in the PD rats compared to the sham rats. The abundance of Selenomonadales and Aeromonadales were increased with the treatment of TPG. Not only at the class and order level, a significant difference in gut microbial was also obviously shown at family (Figure 6G), genus (Figure 6H), and species (Figure 6I) levels among the three groups. Together, these data indicate that PD rats have gut microbial dysbiosis, and TPG can modulate the microbiota compositions to improve gut microbial dysbiosis in PD rats.

**FIGURE 7:**
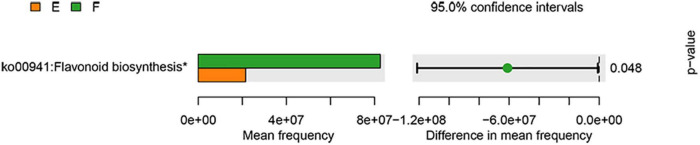
Functional prediction analysis. The data were analyzed by unpaired Student’s *t*-test (*p < 0.05).

Furthermore, given that gut microbial dysbiosis was closely associated with functional changes, functional prediction analysis was also performed in our study (Figure 7). It can be seen that the expression of flavonoid biosynthesis was significantly upregulated by the TPG compared with the PD group. Increasing scientific evidence has shown that flavonoids can have anti-inflammatory properties (24). Considering the role of microbiota-gut-brain axis in PD (25), we next explored the effect of TPG on neuroinflammation in the lesioned striatum.

### Tianqi Pingchan Granule Alleviated Inflammatory Responses

**FIGURE 8:**
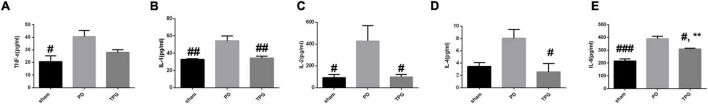
TPG inhibited peripheral proinflammatory cytokine levels. ELISA was used to detect the level of **(A)** TNF-ɑ, **(B)** IL-1, **(C)** IL-2, **(D)** IL-4, and **(E)** IL-6 protein in SD rats of each group. #*p* < 0.05, ##*p* < 0.01, ###*p* < 0.001 vs. PD group; **p < 0.01 sham group.

**FIGURE 9:**
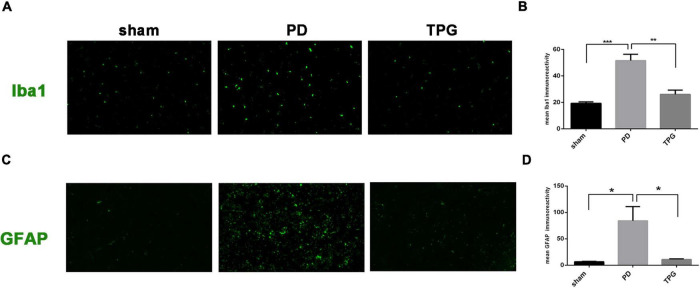
Tianqi Pingchan Granule suppresses neuroinflammation in the lesioned substantia nigra. **(A,B)** Microglia activation was determined by immunohistochemical analysis of Iba1. **(A)**Typical photomicrographs show Iba1 (green)-stained astrocytes in the lesioned substantia nigra. **(B)** Quantitative analysis reveals a significant decrease in the Iba1 staining intensity in TPG rats. *p < 0.05, **p < 0.05 vs. PD group. **(C,D)** Astrocytes activation was determined by immunohistochemical analysis of GFAP. (C) Typical photomicrographs show GFAP (green)-stained astrocytes in the lesioned substantia nigra. **(D)** Quantitative analysis reveals a significant decrease in the GFAP staining intensity in TPG rats. *p < 0.05.

As expected, the serum peripheral inflammatory cytokines in the TPG group were significantly decreased compared to the PD group (Figures 8A–E). In addition, our IHC data revealed that 6-OHDA could significantly increase the expression of glia markers Iba-1 and GFAP in the lesioned substantia nigra of PD rats (Figure 9). The activation of microglia and astrocyte in PD rats in the substantia nigra was largely inhibited by TPG administration.

## Discussion

Parkinson’s disease is a progressive neurodegenerative disease whose global prevalence is rising. Due to its complex underlying mechanisms, single-target drugs are therapeutically less effective in PD management. The multicomponent, multitarget action of TCM exhibits diverse therapeutic effects through the synergistic activity of various pharmacologically active compounds (26). Therefore, treating PD with TCM may be a good choice. However, inferring the mechanisms by which TCM acts is challenging. Network pharmacology has emerged as a valuable tool to analyze the mechanism of the complex components of TCM (27, 28). Based on this, in this study, TPG, a clinically used TCM, was analyzed using a combination of network pharmacological analysis and experimental validation to evaluate its effects and mechanism against PD.

In this study, considering the complexity of the active ingredients in TPG and the diversity of potential regulatory targets in humans, we first collected a summary of active ingredients of TPG meeting the predictive thresholds (DL index ≥ 0.18 and OB ≥ 30%) from TCMSP and identified putative TPG- and PD-related target genes from multiple databases. Then, we obtained TPG-compound-target-PD network to identify PD-related targets in TPG. Furthermore, we constructed the PPI network that identified TNF, IL-1β, and IL-6 as core targets in the context of TPG-mediated PD treatment. The network pharmacology-based findings from KEGG pathway and GO enrichment revealed that TPG -treated PD were mechanically associated with inflammatory response and related signaling pathway, such as NF-κB signaling pathway, T cell receptor signaling pathway, and lipopolysaccharide-mediated signaling pathway. It is well known that the NF-κB and T cell receptor signaling pathway has great effects on peripheral inflammatory response and glia-mediated neuroinflammation (29–32). Consistent with our data, previous studies have shown that the main active ingredients of TPG, such as HQ (30, 33), TM (34), BS (35), and other active derivatives could treat PD through suppression of inflammation response and exerting robust neuroprotection. For example, corynoxine, which is extracted from GT, has been reported to protect dopaminergic neurons through diminishing neuroinflammation in rotenone-induced animal models of PD (36). Calycosin, an isoflavone phytoestrogen isolated from HQ, has been demonstrated to attenuate MPTP-induced PD by suppressing the activation of NF-κB pathways (33). Overall, these data suggest that TPG may treat PD through modulating inflammatory responses.

Additionally, based on the results of network and functional enrichment analysis and given that mounting evidence indicates that gut microbiota community alterations are closely linked to peripheral (37) and central inflammatory responses (23), we first designed animal experiments to test the possibility whether TPG modulates gut microbiota in PD *in vivo*. In this study, the composition of the gut microbiota after TPG treatment was screened using absolute quantification of 16S rRNA. Consistent with previous studies showing that gut microbial dysbiosis exists in patients with PD (38, 39) and mice (23, 40), we verified dysbiosis in the composition of gut microbiota in the 6-OHDA-induced rat model of PD. We demonstrated that gut microbial dysbiosis in PD rats involves significant decreases in class Negativicutes and order Selenomonadales, while significant increases in class Mollicutes, order Enterobacteriales and Mycoplasmatales, are consistent with observations in MPTP-induced PD mice with PD, which have increased abundance of Enterobacteriales (40). In addition, decreases in Negativicutes at the class level have also been reported to be observed in patients with Alzheimer’s disease (41). Strikingly, TPG treatment increased Negativicutes at class level and Selenomonadales at the order level. Our results show that TPG can reverse established microbial dysbiosis. Furthermore, using functional prediction analysis, we found that the expression of flavonoid biosynthesis was significantly upregulated by TPG compared with the PD group. Flavonoids are presented broadly in plants and diets, and are believed to attenuate neuroinflammation in the neurodegenerative process (24, 42).

Considering the prediction revealed by network pharmacological analysis and the close relationship between microbial dysbiosis and peripheral inflammatory responses (37), we further confirmed the critical contribution of TPG to regulate peripheral inflammatory cytokines in PD rats. Cytokines are recognized as important mediators of inflammatory responses (43). Accumulated evidence reveals that peripheral proinflammatory cytokine upregulation in patients with PD, thus suggesting that abnormalities in peripheral immune functions may contribute to the pathogenesis of PD (43). Several studies have reported that inflammatory gene cytokine polymorphisms of TNF-α and IL-1β increase the risk of PD (44, 45). Interestingly, consistent with the network pharmacological analysis, our findings confirmed that oral administration of TPG downregulated serum IL-1, IL-2, IL-4, and IL-6 levels in PD rats, markers of peripheral inflammation.

Finally, beyond the observed alterations in the composition of gut microbiota and peripheral inflammatory cytokines, we further confirmed the role of TPG in regulating central neuroinflammation in PD rats. Numerous studies have shown that alteration in the gut microbiota composition not only affects peripheral immune cell activation but also transfers signals to the central nervous system (CNS) and propagates neuroinflammation in the brain *via* the microbiota-gut-brain axis (22, 23). Similarly, the release of systemic cytokines is critical for CNS effects in response to peripheral immune activation. In this new scenario, it is important to determine whether TPG could not only correct the gut microbial dysbiosis but also alleviate peripheral and central inflammatory responses in PD. Notably, we found that TPG administration reduced the Iba1 and GFAP (markers of microglia and astrocyte activation, respectively) protein expression in the lesioned substantia nigra. Microglia and astrocytes, as key regulators of inflammatory responses in the CNS, respond rapidly to brain microenvironment changes. Taken together, these data suggest that TPG might modify the microenvironment of the central and peripheral immunologic system in PD rats. Our *in vivo* experimental data verify that the strategy of applying network pharmacology to find potential active compounds is reliable and achievable.

## Conclusion

In conclusion, the network pharmacological analysis of TPG identified 73 compounds and 235 target genes associated with PD. TNF-ɑ, IL-1β, IL-6, IL-4, and IL-2 were recognized as essential targets. According to the results of pathway enrichment analysis, we verified that TPG can improve gut microbiota and inhibit the inflammatory response in PD. Network pharmacology strategies can match candidate compounds with potential targets *via* the construction of multicomponent networks. Using network pharmacology and 16S rRNA sequencing-combined approach, the mechanisms of TPG against PD are revealed.

## Data Availability Statement

The raw data supporting the conclusions of this article will be made available by the authors, without undue reservation.

## Ethics Statement

The protocols of this study were reviewed and approved by the Ethical Committee of the Medical School of Shanghai Jiao Tong University (the ethical clearance number: XHEC-NSFC-2019-210).

## Author Contributions

ZGL completed the design of the framework of this manuscript and the experiments. JZ conducted the network pharmacology study. ZHL, SY, YZ, and LS completed the animal experiments. ZHL analyzed the data and prepared the manuscript. NW was responsible for supervision, review, and editing. All authors read and approved the final manuscript.

## Conflict of Interest

The authors declare that the research was conducted in the absence of any commercial or financial relationships that could be construed as a potential conflict of interest.

## Publisher’s Note

All claims expressed in this article are solely those of the authors and do not necessarily represent those of their affiliated organizations, or those of the publisher, the editors and the reviewers. Any product that may be evaluated in this article, or claim that may be made by its manufacturer, is not guaranteed or endorsed by the publisher.

## Footnotes

1
https://old.tcmsp-e.com/index.php

2
http://www.uniprot.org/

3
http://db.idrblab.net/ttd/

4
https://omim.org/

5
https://www.genecards.org/

6
https://go.drugbank.com/

7
https://bioinfogp.cnb.csic.es/tools/venny/index.html

8
http://string-db.org/

9
https://metascape.org/gp/index.html#/citations/

10
http://www.R-project.org/

## Supplementary Material

Supplementary Figure 1 The rarefaction curve for each group.
