# Supplementary figures and images for: Network Pharmacology and Absolute Bacterial Quantification-Combined Approach to Explore the Mechanism of Tianqi Pingchan Granule Against 6-OHDA-Induced Parkinson’s Disease in Rats
Source: Front Nutr. 2022 May 6;9:836500. doi: 10.3389/fnut.2022.836500 (PMC9121100; doi:10.3389/fnut.2022.836500)

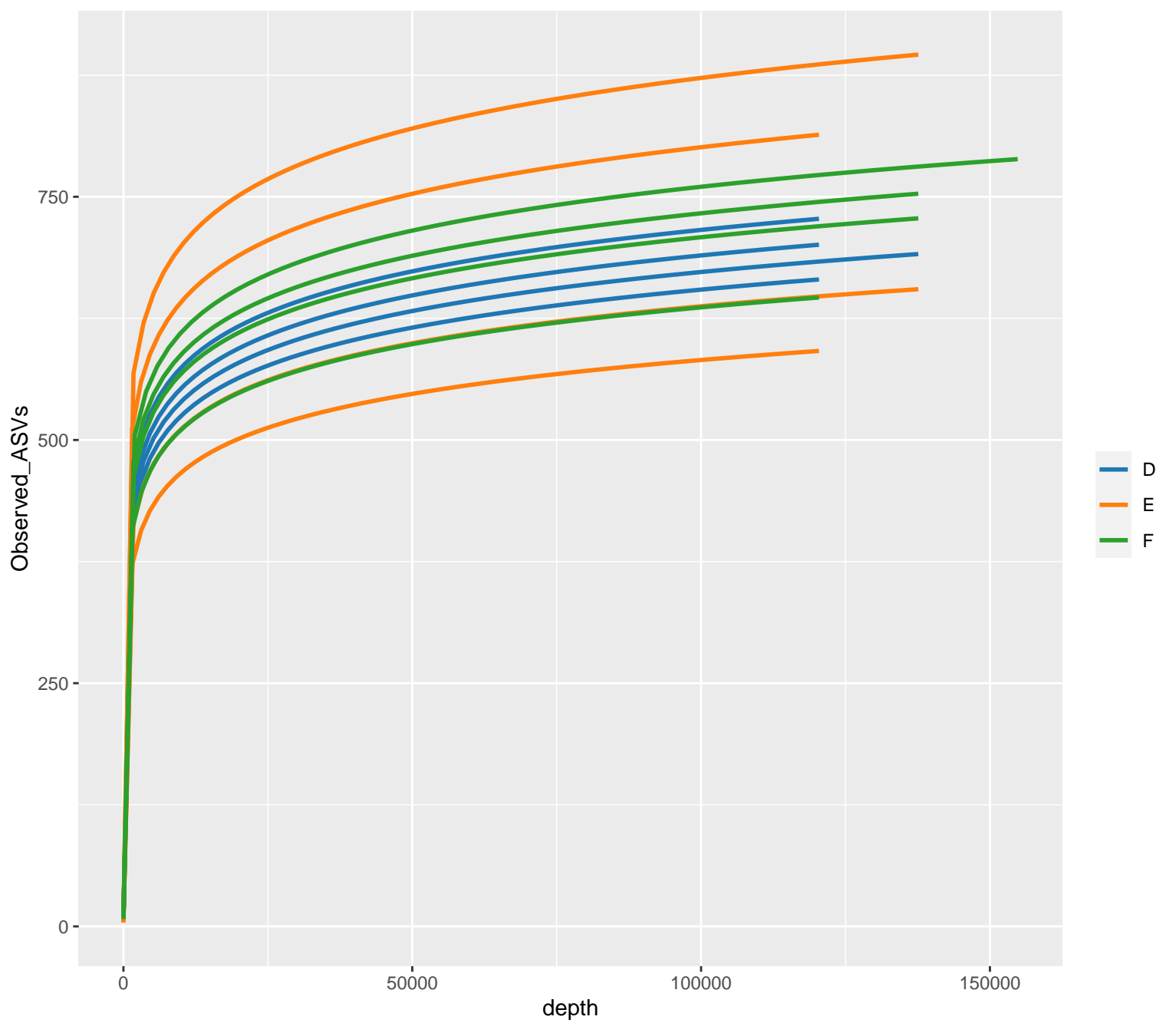

Supplement: Supplementary Figure 1 — The rarefaction curve for each group. [file Image_1.pdf]
