# Supplementary material for: Network Pharmacology and Absolute Bacterial Quantification-Combined Approach to Explore the Mechanism of Tianqi Pingchan Granule Against 6-OHDA-Induced Parkinson’s Disease in Rats
Source: Front Nutr. 2022 May 6;9:836500. doi: 10.3389/fnut.2022.836500 (PMC9121100; doi:10.3389/fnut.2022.836500)
Supplement: Supplementary file 3 [file Table_2.docx]

**Supplementary Table 2. Information for candidate bioactive components of TPG (After eliminating the overlaps).**

| **Number** | **Molecule Name** | **OB (%)** | **DL** | **Herb** |
| --- | --- | --- | --- | --- |
| MOL000211 | Mairin | 55.38 | 0.78 | Astragalus membranaceus |
| MOL000239 | Jaranol | 50.83 | 0.29 | Astragalus membranaceus |
| MOL000296 | hederagenin | 36.91 | 0.75 | Astragalus membranaceus |
| MOL000033 | (3S,8S,9S,10R,13R,14S,17R)-10,13-dimethyl-17-[(2R,5S)-5-propan-2-yloctan-2-yl]-2,3,4,7,8,9,11,12,14,15,16,17-dodecahydro-1H-cyclopenta[a]phenanthren-3-ol | 36.23 | 0.78 | Astragalus membranaceus |
| MOL000354 | isorhamnetin | 49.6 | 0.31 | Astragalus membranaceus |
| MOL000371 | 3,9-di-O-methylnissolin | 53.74 | 0.48 | Astragalus membranaceus |
| MOL000374 | 5'-hydroxyiso-muronulatol-2',5'-di-O-glucoside | 41.72 | 0.69 | Astragalus membranaceus |
| MOL000378 | 7-O-methylisomucronulatol | 74.69 | 0.3 | Astragalus membranaceus |
| MOL000379 | 9,10-dimethoxypterocarpan-3-O-β-D-glucoside | 36.74 | 0.92 | Astragalus membranaceus |
| MOL000380 | (6aR,11aR)-9,10-dimethoxy-6a,11a-dihydro-6H-benzofurano[3,2-c]chromen-3-ol | 64.26 | 0.42 | Astragalus membranaceus |
| MOL000387 | Bifendate | 31.1 | 0.67 | Astragalus membranaceus |
| MOL000392 | formononetin | 69.67 | 0.21 | Astragalus membranaceus |
| MOL000398 | isoflavanone | 109.99 | 0.3 | Astragalus membranaceus |
| MOL000417 | Calycosin | 47.75 | 0.24 | Astragalus membranaceus |
| MOL000422 | kaempferol | 41.88 | 0.24 | Astragalus membranaceus |
| MOL000433 | FA | 68.96 | 0.71 | Astragalus membranaceus |
| MOL000438 | (3R)-3-(2-hydroxy-3,4-dimethoxyphenyl)chroman-7-ol | 67.67 | 0.26 | Astragalus membranaceus |
| MOL000439 | isomucronulatol-7,2'-di-O-glucosiole | 49.28 | 0.62 | Astragalus membranaceus |
| MOL000442 | 1,7-Dihydroxy-3,9-dimethoxy pterocarpene | 39.05 | 0.48 | Astragalus membranaceus |
| MOL000098 | quercetin | 46.43 | 0.28 | Astragalus membranaceus |
| MOL000359 | sitosterol | 36.91 | 0.75 | prepared rhizome of rehmannia |
| MOL000449 | Stigmasterol | 43.83 | 0.76 | prepared rhizome of rehmannia |
| MOL001910 | 11alpha,12alpha-epoxy-3beta-23-dihydroxy-30-norolean-20-en-28,12beta-olide | 64.77 | 0.38 | Radix Paeoniae Alba |
| MOL001918 | paeoniflorgenone | 87.59 | 0.37 | Radix Paeoniae Alba |
| MOL001919 | (3S,5R,8R,9R,10S,14S)-3,17-dihydroxy-4,4,8,10,14-pentamethyl-2,3,5,6,7,9-hexahydro-1H-cyclopenta[a]phenanthrene-15,16-dione | 43.56 | 0.53 | Radix Paeoniae Alba |
| MOL001921 | Lactiflorin | 49.12 | 0.8 | Radix Paeoniae Alba |
| MOL001924 | paeoniflorin | 53.87 | 0.79 | Radix Paeoniae Alba |
| MOL001925 | paeoniflorin_qt | 68.18 | 0.4 | Radix Paeoniae Alba |
| MOL001928 | albiflorin_qt | 66.64 | 0.33 | Radix Paeoniae Alba |
| MOL001930 | benzoyl paeoniflorin | 31.27 | 0.75 | Radix Paeoniae Alba |
| MOL000358 | beta-sitosterol | 36.91 | 0.75 | Radix Paeoniae Alba |
| MOL000492 | (+)-catechin | 54.83 | 0.24 | Radix Paeoniae Alba |
| MOL000073 | ent-Epicatechin | 48.96 | 0.24 | Uncaria rhynchophylla |
| MOL008455 | 3-oxo-22α-hydroxyurs-12-en-27,28-dioc acid | 32.33 | 0.68 | Uncaria rhynchophylla |
| MOL008456 | (3E,4R)-4-(1,3-benzodioxol-5-ylmethyl)-3-[(3,4,5-trimethoxyphenyl)methylidene]oxolan-2-one | 51.78 | 0.65 | Uncaria rhynchophylla |
| MOL008457 | Tetrahydroalstonine | 32.42 | 0.81 | Uncaria rhynchophylla |
| MOL008458 | Angustidine | 51.85 | 0.66 | Uncaria rhynchophylla |
| MOL008460 | geissoschizinc acid | 49.92 | 0.6 | Uncaria rhynchophylla |
| MOL008463 | SMR000232338 | 56.74 | 0.75 | Uncaria rhynchophylla |
| MOL008465 | (E)-16,17-Didehydro-17-methoxy-17,18-seco-3-beta-yohimban-16-carboxylic acid methyl ester | 32.75 | 0.64 | Uncaria rhynchophylla |
| MOL008467 | Rhynchophylline A | 68.68 | 0.69 | Uncaria rhynchophylla |
| MOL008468 | methyl (E)-2-[(2S,3Z,12bS)-3-ethylidene-2,4,6,7,12,12b-hexahydro-1H-indolo[3,2-h]quinolizin-2-yl]-3-methoxyprop-2-enoate | 56.83 | 0.64 | Uncaria rhynchophylla |
| MOL008469 | Rhynchophylline | 41.82 | 0.57 | Uncaria rhynchophylla |
| MOL008470 | SMR000232333 | 78.38 | 0.75 | Uncaria rhynchophylla |
| MOL008471 | Isorhyncophylline | 47.31 | 0.57 | Uncaria rhynchophylla |
| MOL008472 | hirsutasideA | 70.34 | 0.81 | Uncaria rhynchophylla |
| MOL008473 | (E)-2-[(3S,6'S,7'S,8'aS)-6'-ethyl-2-keto-spiro[indoline-3,1'-indolizidine]-7'-yl]-3-methoxy-acrylic acid methyl ester | 57.85 | 0.57 | Uncaria rhynchophylla |
| MOL008474 | (E)-2-[(3R,6'S,7'S,8'aS)-6'-ethyl-2-keto-spiro[indoline-3,1'-indolizidine]-7'-yl]-3-methoxy-acrylic acid methyl ester | 54.47 | 0.57 | Uncaria rhynchophylla |
| MOL008475 | Mitraphyllic acid | 31.7 | 0.7 | Uncaria rhynchophylla |
| MOL008476 | hirsutasideB | 40.21 | 0.8 | Uncaria rhynchophylla |
| MOL008477 | corynoxeine | 57.13 | 0.57 | Uncaria rhynchophylla |
| MOL008478 | methyl (E)-2-[(2S,3R,12bS)-3-vinyl-1,2,3,4,6,7,12,12b-octahydroindolo[3,2-h]quinolizin-2-yl]-3-methoxy-prop-2-enoate | 31.94 | 0.64 | Uncaria rhynchophylla |
| MOL008481 | (1'R,3S,4a'S,5a'S,10a'R)-1'-methyl-2-oxo-1',4a',5',5a',7',8',10',10a'-octahydrospiro[indoline-3,6'-pyrano[3,4-f]indolizine]-4'-carboxylic acid | 105.22 | 0.7 | Uncaria rhynchophylla |
| MOL008482 | (2S,12bR)-methyl 2-((E)-1-oxobut-2-en-2-yl)-1,2,6,7,12,12b-hexahydroindolo[2,3-a]quinolizine-3-carboxylate | 42.07 | 0.6 | Uncaria rhynchophylla |
| MOL008484 | vincoside lactam_qt | 50.81 | 0.82 | Uncaria rhynchophylla |
| MOL008485 | hirsutasideC | 34.27 | 0.75 | Uncaria rhynchophylla |
| MOL008487 | hirsutine | 34.44 | 0.43 | Uncaria rhynchophylla |
| MOL008488 | yohimbine | 46.42 | 0.81 | Uncaria rhynchophylla |
| MOL008489 | delta(sup 18)-Hirsutine | 41.64 | 0.64 | Uncaria rhynchophylla |
| MOL008490 | isocorynantheic acid | 72.36 | 0.6 | Uncaria rhynchophylla |
| MOL008635 | coryincine | 38.27 | 0.81 | Uncaria rhynchophylla |
| MOL000953 | cholesterol | 37.87 | 0.68 | Bombyx Batryticatus |
| MOL005384 | suchilactone | 57.52 | 0.56 | Gastrodia elata Blume |
