# Supplementary material for: Network Pharmacology and Absolute Bacterial Quantification-Combined Approach to Explore the Mechanism of Tianqi Pingchan Granule Against 6-OHDA-Induced Parkinson’s Disease in Rats
Source: Front Nutr. 2022 May 6;9:836500. doi: 10.3389/fnut.2022.836500 (PMC9121100; doi:10.3389/fnut.2022.836500)
Supplement: Supplementary file 4 [file Table_3.docx]

**Supplementary Table 3. Targets of TPG .**

| **Number** | **Molecule name** | **Protein name** | **Gene name** |
| --- | --- | --- | --- |
| 1 | Mairin | Progesterone receptor | PGR |
| 2 | Jaranol | Nitric oxide synthase, inducible | NOS2 |
| 3 | Jaranol | Prostaglandin G/H synthase 1 | PTGS1 |
| 4 | Jaranol | Androgen receptor | AR |
| 5 | Jaranol | Sodium channel protein type 5 subunit alpha | SCN5A |
| 6 | Jaranol | Prostaglandin G/H synthase 2 | PTGS2 |
| 7 | Jaranol | Estrogen receptor beta | ESR2 |
| 8 | Jaranol | Dipeptidyl peptidase IV | DPP4 |
| 9 | Jaranol | Heat shock protein HSP 90 | HSP90AA1 |
| 10 | Jaranol | Serine/threonine-protein kinase Chk1 | CHEK1 |
| 11 | Jaranol | Trypsin-1 | PRSS1 |
| 12 | Jaranol | Nuclear receptor coactivator 2 | NCOA2 |
| 13 | Jaranol | Calmodulin | CALM1 |
| 14 | hederagenin | Muscarinic acetylcholine receptor M3 | CHRM3 |
| 15 | hederagenin | Muscarinic acetylcholine receptor M1 | CHRM1 |
| 16 | hederagenin | Gamma-aminobutyric-acid receptor alpha-2 subunit | GABRA2 |
| 17 | hederagenin | Gamma-aminobutyric-acid receptor alpha-3 subunit | GABRB3 |
| 18 | hederagenin | Muscarinic acetylcholine receptor M2 | CHRM2 |
| 19 | hederagenin | Alpha-1B adrenergic receptor | ADRA1B |
| 20 | hederagenin | Gamma-aminobutyric acid receptor subunit alpha-1 | GABRA1 |
| 21 | hederagenin | Glutamate receptor 2 | GRIA2 |
| 22 | hederagenin | Gamma-aminobutyric-acid receptor subunit alpha-6 | GABRA6 |
| 23 | hederagenin | Gamma-aminobutyric-acid receptor alpha-5 subunit | GABRA5 |
| 24 | hederagenin | Ig gamma-1 chain C region | IGHG1 |
| 25 | hederagenin | Alcohol dehydrogenase 1B | ADH1B |
| 26 | hederagenin | Alcohol dehydrogenase 1C | ADH1C |
| 27 | hederagenin | Lysozyme | LYZ |
| 28 | hederagenin | Retinoic acid receptor RXR-alpha | RXRA |
| 29 | hederagenin | CGMP-inhibited 3',5'-cyclic phosphodiesterase A | PDE3A |
| 30 | hederagenin | Sodium-dependent noradrenaline transporter | SLC6A2 |
| 31 | isorhamnetin | Estrogen receptor | ESR1 |
| 32 | isorhamnetin | Peroxisome proliferator activated receptor gamma | PPARG |
| 33 | isorhamnetin | mRNA of Protein-tyrosine phosphatase, non-receptor type 1 | #N/A |
| 34 | isorhamnetin | Mitogen-activated protein kinase 14 | MAPK14 |
| 35 | isorhamnetin | Glycogen synthase kinase-3 beta | GSK3B |
| 36 | isorhamnetin | Phosphatidylinositol-4,5-bisphosphate 3-kinase catalytic subunit, gamma isoform | PIK3CG |
| 37 | isorhamnetin | Proto-oncogene serine/threonine-protein kinase Pim-1 | PIM1 |
| 38 | isorhamnetin | Cyclin-A2 | CCNA2 |
| 39 | isorhamnetin | Glycogen phosphorylase, muscle form | PYGM |
| 40 | isorhamnetin | Peroxisome proliferator activated receptor delta | PPARD |
| 41 | isorhamnetin | Nuclear receptor coactivator 1 | NCOA1 |
| 42 | isorhamnetin | Coagulation factor VII | F7 |
| 43 | isorhamnetin | Nitric-oxide synthase, endothelial | NOS3 |
| 44 | isorhamnetin | Acetylcholinesterase | ACHE |
| 45 | isorhamnetin | Amine oxidase [flavin-containing] B | MAOB |
| 46 | isorhamnetin | Transcription factor p65 | RELA |
| 47 | isorhamnetin | Xanthine dehydrogenase/oxidase | XDH |
| 48 | isorhamnetin | Neutrophil cytosol factor 1 | NCF1 |
| 49 | isorhamnetin | Oxidized low-density lipoprotein receptor 1 | OLR1 |
| 50 | 3,9-di-O-methylnissolin | Beta-1 adrenergic receptor | ADRB1 |
| 51 | 3,9-di-O-methylnissolin | 5-hydroxytryptamine receptor 3A | HTR3A |
| 52 | 3,9-di-O-methylnissolin | Alpha-2C adrenergic receptor | ADRA2C |
| 53 | 3,9-di-O-methylnissolin | Beta-2 adrenergic receptor | ADRB2 |
| 54 | 3,9-di-O-methylnissolin | Alpha-1D adrenergic receptor | ADRA1D |
| 55 | 3,9-di-O-methylnissolin | Mu-type opioid receptor | OPRM1 |
| 56 | 7-O-methylisomucronulatol | Dopamine D1 receptor | DRD1 |
| 57 | 7-O-methylisomucronulatol | Potassium voltage-gated channel subfamily H member 2 | KCNH2 |
| 58 | 7-O-methylisomucronulatol | Coagulation factor Xa | F10 |
| 59 | 7-O-methylisomucronulatol | Muscarinic acetylcholine receptor M5 | CHRM5 |
| 60 | 7-O-methylisomucronulatol | Muscarinic acetylcholine receptor M4 | CHRM4 |
| 61 | 7-O-methylisomucronulatol | Delta-type opioid receptor | OPRD1 |
| 62 | 7-O-methylisomucronulatol | 5-hydroxytryptamine 2A receptor | HTR2A |
| 63 | 7-O-methylisomucronulatol | Alpha-1A adrenergic receptor | ADRA1A |
| 64 | 7-O-methylisomucronulatol | Sodium-dependent dopamine transporter | SLC6A3 |
| 65 | 7-O-methylisomucronulatol | Sodium-dependent serotonin transporter | SLC6A4 |
| 66 | 7-O-methylisomucronulatol | Retinoic acid receptor RXR-beta | RXRB |
| 67 | 7-O-methylisomucronulatol | Calcium-activated potassium channel subunit alpha 1 | KCNMA1 |
| 68 | 9,10-dimethoxypterocarpan-3-O-β-D-glucoside | DNA topoisomerase II | TOP2A |
| 69 | (6aR,11aR)-9,10-dimethoxy-6a,11a-dihydro-6H-benzofurano[3,2-c]chromen-3-ol | Neuronal acetylcholine receptor protein, alpha-7 chain | CHRNA7 |
| 70 | Bifendate | Vascular endothelial growth factor receptor 2 | KDR |
| 71 | Bifendate | Hepatocyte growth factor receptor | MET |
| 72 | formononetin | cAMP-dependent protein kinase inhibitor alpha | PKIA |
| 73 | formononetin | Beta-lactamase | blaC |
| 74 | formononetin | Transcription factor AP-1 | JUN |
| 75 | formononetin | Peroxisome proliferator-activated receptor gamma | PPARG |
| 76 | formononetin | Interleukin-4 | IL4 |
| 77 | formononetin | NAD-dependent deacetylase sirtuin-1 | SIRT1 |
| 78 | formononetin | ATP synthase subunit beta, mitochondrial | ATP5F1B |
| 79 | formononetin | NADH-ubiquinone oxidoreductase chain 6 | MT-ND6 |
| 80 | formononetin | 3 beta-hydroxysteroid dehydrogenase/Delta 5-->4-isomerase type 2 | HSD3B2 |
| 81 | formononetin | 3 beta-hydroxysteroid dehydrogenase/Delta 5-->4-isomerase type 1 | HSD3B1 |
| 82 | kaempferol | Inhibitor of nuclear factor kappa-B kinase subunit beta | IKBKB |
| 83 | kaempferol | RAC-alpha serine/threonine-protein kinase | AKT1 |
| 84 | kaempferol | Apoptosis regulator Bcl-2 | BCL2 |
| 85 | kaempferol | Apoptosis regulator BAX | BAX |
| 86 | kaempferol | Tumor necrosis factor | TNF |
| 87 | kaempferol | Activator of 90 kDa heat shock protein ATPase homolog 1 | AHSA1 |
| 88 | kaempferol | Caspase-3 | CASP3 |
| 89 | kaempferol | Mitogen-activated protein kinase 8 | MAPK8 |
| 90 | kaempferol | Interstitial collagenase | MMP1 |
| 91 | kaempferol | Signal transducer and activator of transcription 1-alpha/beta | STAT1 |
| 92 | kaempferol | Cell division control protein 2 homolog | CRK2 |
| 93 | kaempferol | Heme oxygenase 1 | HMOX1 |
| 94 | kaempferol | Cytochrome P450 3A4 | CYP3A4 |
| 95 | kaempferol | Cytochrome P450 1A2 | CYP1A2 |
| 96 | kaempferol | Cytochrome P450 1A1 | CYP1A1 |
| 97 | kaempferol | Intercellular adhesion molecule 1 | ICAM1 |
| 98 | kaempferol | E-selectin | SELE |
| 99 | kaempferol | Vascular cell adhesion protein 1 | VCAM1 |
| 100 | kaempferol | Nuclear receptor subfamily 1 group I member 2 | NR1I2 |
| 101 | kaempferol | Cytochrome P450 1B1 | CYP1B1 |
| 102 | kaempferol | Arachidonate 5-lipoxygenase | ALOX5 |
| 103 | kaempferol | Hyaluronan synthase 2 | HAS2 |
| 104 | kaempferol | Glutathione S-transferase P | GSTP1 |
| 105 | kaempferol | Aryl hydrocarbon receptor | AHR |
| 106 | kaempferol | 26S proteasome non-ATPase regulatory subunit 3 | PSMD3 |
| 107 | kaempferol | Solute carrier family 2, facilitated glucose transporter member 4 | SLC2A4 |
| 108 | kaempferol | Nuclear receptor subfamily 1 group I member 3 | NR1I3 |
| 109 | kaempferol | Insulin receptor | INSR |
| 110 | kaempferol | Type I iodothyronine deiodinase | DIO1 |
| 111 | kaempferol | Serine/threonine-protein phosphatase 2B catalytic subunit alpha isoform | PPP3CA |
| 112 | kaempferol | Glutathione S-transferase Mu 1 | GSTM1 |
| 113 | kaempferol | Glutathione S-transferase Mu 2 | GSTM2 |
| 114 | kaempferol | Aldo-keto reductase family 1 member C3 | AKR1C3 |
| 115 | kaempferol | Antileukoproteinase | SLPI |
| 116 | quercetin | Stromelysin-1 | MMP3 |
| 117 | quercetin | Epidermal growth factor receptor | EGFR |
| 118 | quercetin | Vascular endothelial growth factor A | VEGFA |
| 119 | quercetin | G1/S-specific cyclin-D1 | CCND1 |
| 120 | quercetin | Bcl-2-like protein 1 | BCL2L1 |
| 121 | quercetin | Proto-oncogene c-Fos | FOS |
| 122 | quercetin | Cyclin-dependent kinase inhibitor 1 | CDKN1A |
| 123 | quercetin | Eukaryotic translation initiation factor 6 | EIF6 |
| 124 | quercetin | Caspase-9 | CASP9 |
| 125 | quercetin | Urokinase-type plasminogen activator | PLAU |
| 126 | quercetin | 72 kDa type IV collagenase | MMP2 |
| 127 | quercetin | Matrix metalloproteinase-9 | MMP9 |
| 128 | quercetin | Mitogen-activated protein kinase 1 | MAPK1 |
| 129 | quercetin | Interleukin-10 | IL10 |
| 130 | quercetin | Pro-epidermal growth factor | EGF |
| 131 | quercetin | Retinoblastoma-associated protein | RB1 |
| 132 | quercetin | Interleukin-6 | IL6 |
| 133 | quercetin | Cyclin-dependent kinase inhibitor 2A, isoforms 1/2/3 | CDKN2A |
| 134 | quercetin | Cellular tumor antigen p53 | TP53 |
| 135 | quercetin | ETS domain-containing protein Elk-1 | ELK1 |
| 136 | quercetin | NF-kappa-B inhibitor alpha | NFKBIA |
| 137 | quercetin | NADPH--cytochrome P450 reductase | POR |
| 138 | quercetin | Ornithine decarboxylase | ODC1 |
| 139 | quercetin | Caspase-8 | CASP8 |
| 140 | quercetin | DNA topoisomerase 1 | TOP1 |
| 141 | quercetin | RAF proto-oncogene serine/threonine-protein kinase | RAF1 |
| 142 | quercetin | Superoxide dismutase [Cu-Zn] | SOD1 |
| 143 | quercetin | Protein kinase C alpha type | PRKCA |
| 144 | quercetin | Hypoxia-inducible factor 1-alpha | HIF1A |
| 145 | quercetin | Protein CBFA2T1 | RUNX1T1 |
| 146 | quercetin | Probable E3 ubiquitin-protein ligase HERC5 | HERC5 |
| 147 | quercetin | 78 kDa glucose-regulated protein | HSPA5 |
| 148 | quercetin | Receptor tyrosine-protein kinase erbB-2 | ERBB2 |
| 149 | quercetin | Acetyl-CoA carboxylase 1 | ACACA |
| 150 | quercetin | Caveolin-1 | CAV1 |
| 151 | quercetin | Myc proto-oncogene protein | MYC |
| 152 | quercetin | Tissue factor | F3 |
| 153 | quercetin | Gap junction alpha-1 protein | GJA1 |
| 154 | quercetin | Interleukin-1 beta | IL1B |
| 155 | quercetin | C-C motif chemokine 2 | CCL2 |
| 156 | quercetin | Prostaglandin E2 receptor EP3 subtype | PTGER3 |
| 157 | quercetin | Interleukin-8 | CXCL8 |
| 158 | quercetin | Protein kinase C beta type | PRKCB |
| 159 | quercetin | Baculoviral IAP repeat-containing protein 5 | BIRC5 |
| 160 | quercetin | Dual oxidase 2 | DUOX2 |
| 161 | quercetin | Nitric oxide synthase, endothelial | NOS3 |
| 162 | quercetin | Heat shock protein beta-1 | HSPB1 |
| 163 | quercetin | Transforming growth factor beta-1 | TGFB1 |
| 164 | quercetin | Estrogen sulfotransferase | SULT1E1 |
| 165 | quercetin | Maltase-glucoamylase, intestinal | MGAM |
| 166 | quercetin | Interleukin-2 | IL2 |
| 167 | quercetin | G2/mitotic-specific cyclin-B1 | CCNB1 |
| 168 | quercetin | Tissue-type plasminogen activator | PLAT |
| 169 | quercetin | Thrombomodulin | THBD |
| 170 | quercetin | Plasminogen activator inhibitor 1 | SERPINE1 |
| 171 | quercetin | Collagen alpha-1(I) chain | COL1A1 |
| 172 | quercetin | Interferon gamma | IFNG |
| 173 | quercetin | Phosphatidylinositol-3,4,5-trisphosphate 3-phosphatase and dual-specificity protein phosphatase PTEN | PTEN |
| 174 | quercetin | Interleukin-1 alpha | IL1A |
| 175 | quercetin | Myeloperoxidase | MPO |
| 176 | quercetin | DNA topoisomerase 2-alpha | TOP2A |
| 177 | quercetin | ATP-binding cassette sub-family G member 2 | ABCG2 |
| 178 | quercetin | Nuclear factor erythroid 2-related factor 2 | NFE2L2 |
| 179 | quercetin | NAD(P)H dehydrogenase [quinone] 1 | NQO1 |
| 180 | quercetin | Poly [ADP-ribose] polymerase 1 | PARP1 |
| 181 | quercetin | Collagen alpha-1(III) chain | COL3A1 |
| 182 | quercetin | DNA gyrase subunit B | gyrB |
| 183 | quercetin | C-X-C motif chemokine 11 | CXCL11 |
| 184 | quercetin | C-X-C motif chemokine 2 | CXCL2 |
| 185 | quercetin | DDB1- and CUL4-associated factor 5 | DCAF5 |
| 186 | quercetin | Serine/threonine-protein kinase Chk2 | CHEK2 |
| 187 | quercetin | Claudin-4 | CLDN4 |
| 188 | quercetin | Peroxisome proliferator-activated receptor alpha | PPARA |
| 189 | quercetin | Peroxisome proliferator-activated receptor delta | PPARD |
| 190 | quercetin | Heat shock factor protein 1 | HSF1 |
| 191 | quercetin | C-reactive protein | CRP |
| 192 | quercetin | C-X-C motif chemokine 10 | CXCL10 |
| 193 | quercetin | Inhibitor of nuclear factor kappa-B kinase subunit alpha | CHUK |
| 194 | quercetin | Osteopontin | SPP1 |
| 195 | quercetin | Runt-related transcription factor 2 | RUNX2 |
| 196 | quercetin | Ras association domain-containing protein 1 | RASSF1 |
| 197 | quercetin | Transcription factor E2F1 | E2F1 |
| 198 | quercetin | Transcription factor E2F2 | E2F2 |
| 199 | quercetin | Prostatic acid phosphatase | ACP3 |
| 200 | quercetin | Cathepsin D | CTSD |
| 201 | quercetin | Insulin-like growth factor-binding protein 3 | IGFBP3 |
| 202 | quercetin | Insulin-like growth factor II | IGF2 |
| 203 | quercetin | CD40 ligand | CD40LG |
| 204 | quercetin | Interferon regulatory factor 1 | IRF1 |
| 205 | quercetin | Receptor tyrosine-protein kinase erbB-3 | ERBB3 |
| 206 | quercetin | Serum paraoxonase/arylesterase 1 | PON1 |
| 207 | quercetin | Procollagen C-endopeptidase enhancer 1 | PCOLCE |
| 208 | quercetin | Puromycin-sensitive aminopeptidase | NPEPPS |
| 209 | quercetin | Hexokinase-2 | HK2 |
| 210 | quercetin | Homeobox protein Nkx-3.1 | NKX3-1 |
| 211 | quercetin | Ras GTPase-activating protein 1 | RASA1 |
| 212 | sitosterol | Mineralocorticoid receptor | NR3C2 |
| 213 | Stigmasterol | Alpha-2A adrenergic receptor | ADRA2A |
| 214 | Stigmasterol | Leukotriene A-4 hydrolase | LTA4H |
| 215 | Stigmasterol | Amine oxidase [flavin-containing] A | MAOA |
| 216 | Stigmasterol | Chymotrypsinogen B | CTRB1 |
| 217 | paeoniflorin | Monocyte differentiation antigen CD14 | CD14 |
| 218 | paeoniflorin | Lipopolysaccharide-binding protein | LBP |
| 219 | beta-sitosterol | Neuronal acetylcholine receptor subunit alpha-2 | CHRNA2 |
| 220 | beta-sitosterol | Microtubule-associated protein 2 | MAP2 |
| 221 | (+)-catechin | Catalase | CAT |
| 222 | (3E,4R)-4-(1,3-benzodioxol-5-ylmethyl)-3-[(3,4,5-trimethoxyphenyl)methylidene]oxolan-2-one | cAMP and cAMP-inhibited cGMP 3',5'-cyclic phosphodiesterase 10A | PDE10A |
| 223 | geissoschizinc acid | Carbonic anhydrase II | CA2 |
| 224 | (E)-16,17-Didehydro-17-methoxy-17,18-seco-3-beta-yohimban-16-carboxylic acid methyl ester | 5-hydroxytryptamine 2C receptor | HTR2C |
| 225 | (E)-16,17-Didehydro-17-methoxy-17,18-seco-3-beta-yohimban-16-carboxylic acid methyl ester | Alpha-2B adrenergic receptor | ADRA2B |
| 226 | (E)-16,17-Didehydro-17-methoxy-17,18-seco-3-beta-yohimban-16-carboxylic acid methyl ester | Beta-secretase | BACE1 |
| 227 | Isorhyncophylline | D(2) dopamine receptor | DRD2 |
| 228 | hirsutine | D(1B) dopamine receptor | DRD5 |
| 229 | hirsutine | Kappa-type opioid receptor | OPRK1 |
| 230 | yohimbine | D(4) dopamine receptor | DRD4 |
| 231 | yohimbine | D(3) dopamine receptor | DRD3 |
| 232 | yohimbine | Midkine | MDK |
| 233 | yohimbine | Beta-nerve growth factor | NGF |
| 234 | yohimbine | Lipoprotein lipase | LPL |
| 235 | delta(sup 18)-Hirsutine | 5-hydroxytryptamine 7 receptor | HTR7 |
