# Supplementary material for: Network Pharmacology and Absolute Bacterial Quantification-Combined Approach to Explore the Mechanism of Tianqi Pingchan Granule Against 6-OHDA-Induced Parkinson’s Disease in Rats
Source: Front Nutr. 2022 May 6;9:836500. doi: 10.3389/fnut.2022.836500 (PMC9121100; doi:10.3389/fnut.2022.836500)
Supplement: Supplementary file 5 [file Table_4.docx]

**Supplementary Table 4. PD-related target genes.**

| **Number** | **Targets** |
| --- | --- |
| 1 | DRD2 |
| 2 | M |
| 3 | DDC |
| 4 | MAOB |
| 5 | SLC22A2 |
| 6 | SLC22A1 |
| 7 | GRIN3A |
| 8 | CHRNA7 |
| 9 | CHRNA4 |
| 10 | CHRNA3 |
| 11 | DRD1 |
| 12 | DRD4 |
| 13 | DRD3 |
| 14 | DRD5 |
| 15 | ADRA2C |
| 16 | ADRA2B |
| 17 | HTR2C |
| 18 | HTR1A |
| 19 | HTR2A |
| 20 | HTR2B |
| 21 | ADRA2A |
| 22 | HTR1D |
| 23 | HTR1B |
| 24 | COMT |
| 25 | SULT1A1 |
| 26 | SULT1A2 |
| 27 | SULT1A3 |
| 28 | SULT1E1 |
| 29 | SULT1B1 |
| 30 | CYP2B6 |
| 31 | CYP2C8 |
| 32 | CYP3A4 |
| 33 | CYP3A5 |
| 34 | UGT1A1 |
| 35 | ALB |
| 36 | SLC18A2 |
| 37 | ADRA1A |
| 38 | ADRA1B |
| 39 | ADRA1D |
| 40 | HTR7 |
| 41 | ABCB1 |
| 42 | PAH |
| 43 | ADRB1 |
| 44 | ADRB2 |
| 45 | ADRB3 |
| 46 | SLC16A10 |
| 47 | SLC6A2 |
| 48 | UGT1A9 |
| 49 | CYP2D6 |
| 50 | CYP1A1 |
| 51 | CYP1A2 |
| 52 | CYP2C9 |
| 53 | CYP2C18 |
| 54 | ADORA2A |
| 55 | ADORA1 |
| 56 | ORM1 |
| 57 | ABCG2 |
| 58 | SLCO1B1 |
| 59 | SLCO1B3 |
| 60 | SLC22A6 |
| 61 | POU2F2 |
| 62 | SLC47A1 |
| 63 | SLC47A2 |
| 64 | SLC15A1 |
| 65 | SLC7A5 |
| 66 | SLC7A8 |
| 67 | CYP2C19 |
| 68 | SLC22A3 |
| 69 | CHRM1 |
| 70 | BCHE |
| 71 | CHRM2 |
| 72 | HRH1 |
| 73 | CHRM3 |
| 74 | CHRM4 |
| 75 | CHRM5 |
| 76 | HTR1E |
| 77 | HTR3A |
| 78 | HTR6 |
| 79 | CYP3A7 |
| 80 | BCL2 |
| 81 | MAOA |
| 82 | SLCO3A1 |
| 83 | CYP2E1 |
| 84 | CYP2A6 |
| 85 | UGT1A10 |
| 86 | UGT1A3 |
| 87 | UGT1A4 |
| 88 | UGT1A6 |
| 89 | UGT1A7 |
| 90 | UGT1A8 |
| 91 | UGT2B15 |
| 92 | UGT2B4 |
| 93 | UGT2B7 |
| 94 | CYP3A43 |
| 95 | ORM2 |
| 96 | D2R |
| 97 | MAO-B |
| 98 | GRIA |
| 99 | OPRS1 |
| 100 | D3R |
| 101 | D4R |
| 102 | SNDR |
| 103 | H1R |
| 104 | AKT1 |
| 105 | PDE1 |
| 106 | TAAR1 |
| 107 | mGluR5 |
| 108 | GABRG3 |
| 109 | AChE |
| 110 | GRIA1 |
| 111 | GRIA2 |
| 112 | GRIA3 |
| 113 | GRIA4 |
| 114 | ACACB |
| 115 | Malaria ACC1 |
| 116 | SNCA |
| 117 | nAChR |
| 118 | SRC |
| 119 | NQO |
| 120 | VAMP |
| 121 | DAT |
| 122 | D1R |
| 123 | MT-ND3 |
| 124 | NMDAR |
| 125 | NMDAR2B |
| 126 | PREP |
| 127 | HTR4 |
| 128 | GLUL |
| 129 | KCNQ |
| 130 | OPR |
| 131 | PDE4D |
| 132 | PDE4B |
| 133 | PDE4A |
| 134 | Raf mRNA |
| 135 | BRAF mRNA |
| 136 | SERT |
| 137 | Cav |
| 138 | PRKC |
| 139 | ADORA2B |
| 140 | APCS |
| 141 | GCH1 |
| 142 | TH |
| 143 | GSK-3B |
| 144 | MPO |
| 145 | NaC |
| 146 | COX |
| 147 | FKBP1A |
| 148 | NET |
| 149 | NGFR |
| 150 | GS |
| 151 | TRH |
| 152 | CHRNA4/B2 |
| 153 | LRRK2 |
| 154 | 5HTR |
| 155 | UGCG |
| 156 | MAO-A |
| 157 | MAO |
| 158 | T-CaC |
| 159 | ADR |
| 160 | GABAR |
| 161 | PLEKHG5 |
| 162 | PARK7 |
| 163 | KIF1B |
| 164 | MTHFR |
| 165 | MFN2 |
| 166 | PINK1 |
| 167 | ECE1 |
| 168 | RHCE |
| 169 | YARS1 |
| 170 | FOXD3 |
| 171 | DNAJC6 |
| 172 | IL23R |
| 173 | TNNI3K |
| 174 | ABCA4 |
| 175 | AGL |
| 176 | DBT |
| 177 | ATP1A1 |
| 178 | NOTCH2NLC |
| 179 | ECM1 |
| 180 | ZNF687 |
| 181 | MUC1 |
| 182 | GBA |
| 183 | LMNA |
| 184 | COPA |
| 185 | MPZ |
| 186 | NCF2 |
| 187 | INAVA |
| 188 | IL10 |
| 189 | TLR5 |
| 190 | PSEN2 |
| 191 | ADAM17 |
| 192 | MPV17 |
| 193 | ABCG8 |
| 194 | HTRA2 |
| 195 | ZAP70 |
| 196 | HOXD10 |
| 197 | PDE11A |
| 198 | BMPR2 |
| 199 | CTLA4 |
| 200 | FARSB |
| 201 | IRS1 |
| 202 | SP110 |
| 203 | GIGYF2 |
| 204 | ATG16L1 |
| 205 | SAG |
| 206 | PDCD1 |
| 207 | CAV3 |
| 208 | CX3CR1 |
| 209 | GBE1 |
| 210 | POGLUT1 |
| 211 | RAB7A |
| 212 | ATP2C1 |
| 213 | DZIP1L |
| 214 | GYG1 |
| 215 | MME |
| 216 | GNB4 |
| 217 | EIF4G1 |
| 218 | DNAJB11 |
| 219 | HTT |
| 220 | PROM1 |
| 221 | UCHL1 |
| 222 | PHOX2B |
| 223 | PKD2 |
| 224 | PPM1K |
| 225 | ADH1C |
| 226 | TRIM2 |
| 227 | GDNF |
| 228 | HEXB |
| 229 | PDE8B |
| 230 | APC |
| 231 | SAR1B |
| 232 | HARS1 |
| 233 | SH3TC2 |
| 234 | IRGM |
| 235 | SQSTM1 |
| 236 | HFE |
| 237 | DHX16 |
| 238 | HLA-DQA1 |
| 239 | HLA-DQB1 |
| 240 | HLA-DPB1 |
| 241 | PKHD1 |
| 242 | SLC17A5 |
| 243 | ELOVL4 |
| 244 | BCKDHB |
| 245 | SEC63 |
| 246 | FIG4 |
| 247 | ENPP1 |
| 248 | LPA |
| 249 | PRKN |
| 250 | TBP |
| 251 | IL6 |
| 252 | HNRNPA2B1 |
| 253 | GARS1 |
| 254 | SFRP4 |
| 255 | PGAM2 |
| 256 | EGFR |
| 257 | CHCHD2 |
| 258 | NCF1 |
| 259 | HSPB1 |
| 260 | CD36 |
| 261 | ABCB4 |
| 262 | PON1 |
| 263 | PON2 |
| 264 | ARPC1B |
| 265 | IRF5 |
| 266 | NOS3 |
| 267 | PRKAG2 |
| 268 | GATA4 |
| 269 | NEFL |
| 270 | JPH1 |
| 271 | GDAP1 |
| 272 | PMP2 |
| 273 | TNFRSF11B |
| 274 | TG |
| 275 | NDRG1 |
| 276 | ZFAT |
| 277 | SLURP1 |
| 278 | VCP |
| 279 | ABCA1 |
| 280 | CRB2 |
| 281 | LRSAM1 |
| 282 | GLE1 |
| 283 | SURF1 |
| 284 | NOTCH1 |
| 285 | DHTKD1 |
| 286 | PHYH |
| 287 | RET |
| 288 | EGR2 |
| 289 | SLC25A16 |
| 290 | PSAP |
| 291 | PLAU |
| 292 | ACTA2 |
| 293 | LIPA |
| 294 | PDZD7 |
| 295 | CALHM1 |
| 296 | PNPLA2 |
| 297 | SMPD1 |
| 298 | SBF2 |
| 299 | LDHA |
| 300 | MAPT |
| 301 | SLC6A3 |
| 302 | APOE |
| 303 | SNCAIP |
| 304 | SYNJ1 |
| 305 | VPS35 |
| 306 | ATP13A2 |
| 307 | APP |
| 308 | PSEN1 |
| 309 | PLA2G6 |
| 310 | PRNP |
| 311 | FBXO7 |
| 312 | BDNF |
| 313 | SOD1 |
| 314 | TNF |
| 315 | POLG |
| 316 | ATXN2 |
| 317 | DNAJC13 |
| 318 | NR4A2 |
| 319 | VPS13C |
| 320 | TAF1 |
| 321 | CAT |
| 322 | TARDBP |
| 323 | GFAP |
| 324 | SNCB |
| 325 | MT-ND1 |
| 326 | DYNC1H1 |
| 327 | CHAT |
| 328 | MT-ND5 |
| 329 | TTR |
| 330 | RAB39B |
| 331 | PRODH |
| 332 | NDUFS4 |
| 333 | MTOR |
| 334 | HRAS |
| 335 | MIR132 |
| 336 | CP |
| 337 | C9orf72 |
| 338 | PTEN |
| 339 | NGF |
| 340 | ATXN3 |
| 341 | CTSD |
| 342 | DCTN1 |
| 343 | SOD2 |
| 344 | IL1B |
| 345 | TP53 |
| 346 | CACNA1A |
| 347 | GAA |
| 348 | SLC6A4 |
| 349 | CASP8 |
| 350 | GRN |
| 351 | HCRT |
| 352 | HMOX1 |
| 353 | NFE2L2 |
| 354 | SORL1 |
| 355 | MYC |
| 356 | INS |
| 357 | MIR21 |
| 358 | TOR1A |
| 359 | ACE |
| 360 | GRIN2B |
| 361 | COQ2 |
| 362 | GSR |
| 363 | FUS |
| 364 | DNM1L |
| 365 | FGF20 |
| 366 | HUWE1 |
| 367 | ACTB |
| 368 | BAX |
| 369 | TYR |
| 370 | PANK2 |
| 371 | IFNG |
| 372 | PODXL |
| 373 | PITX3 |
| 374 | TFAM |
| 375 | UBE2L3 |
| 376 | VEGFA |
| 377 | PINK1-AS |
| 378 | TREM2 |
| 379 | SOX2 |
| 380 | ATXN7 |
| 381 | NTRK2 |
| 382 | MIR22 |
| 383 | CD40LG |
| 384 | NRTN |
| 385 | SERPINA3 |
| 386 | SYP |
| 387 | UBB |
| 388 | IGF2R |
| 389 | MAPK10 |
| 390 | CHMP2B |
| 391 | TLR4 |
| 392 | DYRK1A |
| 393 | ATXN1 |
| 394 | PDYN |
| 395 | ATP1A3 |
| 396 | CDC42 |
| 397 | TMEM106B |
| 398 | H19 |
| 399 | C19orf12 |
| 400 | ATXN8OS |
| 401 | WASHC5 |
| 402 | FGF8 |
| 403 | RPS27A |
| 404 | STX1B |
| 405 | BAP1 |
| 406 | MT-ND6 |
| 407 | IGF1 |
| 408 | NCSTN |
| 409 | OTX2 |
| 410 | SPR |
| 411 | SLC11A2 |
| 412 | FTL |
| 413 | LMX1B |
| 414 | GRM1 |
| 415 | NTF4 |
| 416 | ATP6AP2 |
| 417 | PARK16 |
| 418 | UBE3A |
| 419 | STUB1 |
| 420 | CACNA1D |
| 421 | CCR6 |
| 422 | ATXN10 |
| 423 | ATP7B |
| 424 | LAMP2 |
| 425 | OGDH |
| 426 | PACRG |
| 427 | TLR2 |
| 428 | ERCC6 |
| 429 | PSENEN |
| 430 | ACTG1 |
| 431 | NPC1 |
| 432 | APOA1 |
| 433 | CTNNB1 |
| 434 | FOXO1 |
| 435 | GLUD2 |
| 436 | CHM |
| 437 | MSX1 |
| 438 | PARK10 |
| 439 | FMR1 |
| 440 | HSPA9 |
| 441 | CASP3 |
| 442 | LOC106627981 |
| 443 | SPG11 |
| 444 | MCCC1 |
| 445 | FAS |
| 446 | C1QBP |
| 447 | SCARB2 |
| 448 | LMX1A |
| 449 | UBA1 |
| 450 | APPL1 |
| 451 | FA2H |
| 452 | PDGFRB |
| 453 | MT-ATP6 |
| 454 | MAF |
| 455 | SLC2A1 |
| 456 | PARK21 |
| 457 | ABCA7 |
| 458 | GRIK2 |
| 459 | CDK5 |
| 460 | CRYAA |
| 461 | KCNC3 |
| 462 | RAB11A |
| 463 | DDOST |
| 464 | PARK12 |
| 465 | STAT3 |
| 466 | MT-ND4 |
| 467 | CTSB |
| 468 | JAG1 |
| 469 | NOD2 |
| 470 | MIR433 |
| 471 | MIR331 |
| 472 | PRKRA |
| 473 | TSC2 |
| 474 | MIR133B |
| 475 | MYH7 |
| 476 | SNAP25 |
| 477 | NQO2 |
| 478 | WASHC4 |
| 479 | NDUFV2 |
| 480 | ARSA |
| 481 | MT-CO1 |
| 482 | GSK3B |
| 483 | CRH |
| 484 | BMP2 |
| 485 | SCN5A |
| 486 | SGCE |
| 487 | TNNT2 |
| 488 | AR |
| 489 | CDKN2A |
| 490 | PIK3CA |
| 491 | SNX3 |
| 492 | CYCS |
| 493 | TMEM230 |
| 494 | RYR1 |
| 495 | NOS2 |
| 496 | SNX27 |
| 497 | DBH |
| 498 | SNCG |
| 499 | RAB40AL |
| 500 | TNFRSF1A |
| 501 | REN |
| 502 | MAPK1 |
| 503 | THAP1 |
| 504 | SERPINA1 |
| 505 | PICALM |
| 506 | TNNI3 |
| 507 | NTRK1 |
| 508 | STAT1 |
| 509 | RYR2 |
| 510 | MBP |
| 511 | GAPDH |
| 512 | ERBB2 |
| 513 | MYH6 |
| 514 | PPARG |
| 515 | TBX5 |
| 516 | FASLG |
| 517 | LRP5 |
| 518 | MT-CYB |
| 519 | PDGFB |
| 520 | NR1H4 |
| 521 | WDR45 |
| 522 | TF |
| 523 | BACE1 |
| 524 | IGHMBP2 |
| 525 | EGF |
| 526 | KIF5A |
| 527 | COASY |
| 528 | HSPA4 |
| 529 | KRAS |
| 530 | MT-TK |
| 531 | CRYAB |
| 532 | NOS1 |
| 533 | CST3 |
| 534 | MAPK8 |
| 535 | GJA1 |
| 536 | JUP |
| 537 | TBK1 |
| 538 | CDH1 |
| 539 | H2AC18 |
| 540 | SMN1 |
| 541 | REST |
| 542 | FGFR1 |
| 543 | MAPK14 |
| 544 | MIRLET7I |
| 545 | MIR126 |
| 546 | HEXA |
| 547 | TWNK |
| 548 | KIT |
| 549 | DSC2 |
| 550 | APOB |
| 551 | FN1 |
| 552 | CASP9 |
| 553 | SLC1A3 |
| 554 | JAK2 |
| 555 | SLC25A4 |
| 556 | PNKD |
| 557 | RBFOX3 |
| 558 | GPR37 |
| 559 | PITX2 |
| 560 | CXCL8 |
| 561 | HPCA |
| 562 | CSF1R |
| 563 | KARS1 |
| 564 | G6PD |
| 565 | HLA-DRB1 |
| 566 | MYBPC3 |
| 567 | SLC30A10 |
| 568 | KCNQ1 |
| 569 | TGFBR2 |
| 570 | MIR17 |
| 571 | KLK6 |
| 572 | CXCR4 |
| 573 | NEK1 |
| 574 | HSP90AA1 |
| 575 | SCN1A |
| 576 | TPM3 |
| 577 | DSC3 |
| 578 | NF1 |
| 579 | NKX2-5 |
| 580 | MCOLN1 |
| 581 | SLC39A14 |
| 582 | GJB1 |
| 583 | CACNA1C |
| 584 | GLA |
| 585 | MT-ATP8 |
| 586 | CNR1 |
| 587 | JUN |
| 588 | MAPK3 |
| 589 | MECP2 |
| 590 | BSCL2 |
| 591 | PRDM10 |
| 592 | CHCHD10 |
| 593 | NEFH |
| 594 | SYT11 |
| 595 | SLC1A2 |
| 596 | ERBB4 |
| 597 | VDAC1 |
| 598 | NPC2 |
| 599 | COL4A1 |
| 600 | HNRNPA1 |
| 601 | PVALB |
| 602 | KCNH2 |
| 603 | SCN9A |
| 604 | MAP2K1 |
| 605 | PTPN11 |
| 606 | DKK1 |
| 607 | PYGM |
| 608 | SERPINC1 |
| 609 | SCN8A |
| 610 | IGF2 |
| 611 | LPL |
| 612 | VIM |
| 613 | SI |
| 614 | CD40 |
| 615 | ATN1 |
| 616 | CREBBP |
| 617 | NTF3 |
| 618 | TGFB1 |
| 619 | ATM |
| 620 | ATP2B3 |
| 621 | TGM2 |
| 622 | ESR2 |
| 623 | TMEM67 |
| 624 | CFH |
| 625 | DCAF17 |
| 626 | ANK2 |
| 627 | ACTC1 |
| 628 | HLA-DRA |
| 629 | SHH |
| 630 | GAD1 |
| 631 | OPTN |
| 632 | CLU |
| 633 | MT-ND2 |
| 634 | NFKB1 |
| 635 | U2AF1 |
| 636 | GFM1 |
| 637 | JPH3 |
| 638 | PARP1 |
| 639 | TERT |
| 640 | NRAS |
| 641 | GNAL |
| 642 | BECN1 |
| 643 | VPS26A |
| 644 | NAT2 |
| 645 | NDUFV1 |
| 646 | RD3 |
| 647 | P4HB |
| 648 | CCND1 |
| 649 | SMN2 |
| 650 | NDUFS3 |
| 651 | MMP9 |
| 652 | MT-CO2 |
| 653 | SMAD3 |
| 654 | CAPN1 |
| 655 | HSPA8 |
| 656 | STH |
| 657 | TSC1 |
| 658 | TPH1 |
| 659 | MAP2 |
| 660 | RNF19A |
| 661 | GLI3 |
| 662 | INPP5E |
| 663 | GRIN2A |
| 664 | NQO1 |
| 665 | GAK |
| 666 | SETX |
| 667 | GRM5 |
| 668 | IREB2 |
| 669 | MIR125A |
| 670 | EP300 |
| 671 | MDM2 |
| 672 | BRCA2 |
| 673 | TSPO |
| 674 | RAB29 |
| 675 | MIR9-1 |
| 676 | MIR142 |
| 677 | HGF |
| 678 | CALB1 |
| 679 | VPS13A |
| 680 | CCL3 |
| 681 | RELN |
| 682 | CNTF |
| 683 | IGF1R |
| 684 | MGMT |
| 685 | FXN |
| 686 | IL4 |
| 687 | BRCA1 |
| 688 | MSH2 |
| 689 | MIR146A |
| 690 | GDF6 |
| 691 | IDUA |
| 692 | AUP1 |
| 693 | PARL |
| 694 | ANO3 |
| 695 | GALC |
| 696 | TAC1 |
| 697 | DMD |
| 698 | CHI3L1 |
| 699 | CLN3 |
| 700 | DMPK |
| 701 | PPARGC1A |
| 702 | LINGO1 |
| 703 | MIR24-1 |
| 704 | MIR34A |
| 705 | AIMP2 |
| 706 | NHLRC1 |
| 707 | CCL2 |
| 708 | PAX2 |
| 709 | ITPR1 |
| 710 | IL1A |
| 711 | AIF1 |
| 712 | GYS1 |
| 713 | TK2 |
| 714 | PHB |
| 715 | NTS |
| 716 | UBQLN2 |
| 717 | CFTR |
| 718 | SLC26A4 |
| 719 | LAMP1 |
| 720 | IL2 |
| 721 | DES |
| 722 | ADNP |
| 723 | RRM2B |
| 724 | KMT2B |
| 725 | SDHB |
| 726 | TUBA1B |
| 727 | PAX6 |
| 728 | EPM2A |
| 729 | ALS2 |
| 730 | MIR124-1 |
| 731 | BST1 |
| 732 | F2 |
| 733 | SCN2A |
| 734 | BMP4 |
| 735 | HLA-DRB5 |
| 736 | DNMT1 |
| 737 | EPHA3 |
| 738 | NOTCH2 |
| 739 | NRG1 |
| 740 | TRPM7 |
| 741 | STXBP1 |
| 742 | SLC20A2 |
| 743 | TIA1 |
| 744 | IL17A |
| 745 | CAV1 |
| 746 | GM2A |
| 747 | S100B |
| 748 | TUBA4A |
| 749 | CBS |
| 750 | SPTLC1 |
| 751 | NDUFA1 |
| 752 | PDGFRA |
| 753 | PCDH19 |
| 754 | RAB5A |
| 755 | GLUD1 |
| 756 | SLC11A1 |
| 757 | MIR223 |
| 758 | HP |
| 759 | ADAM10 |
| 760 | DLG4 |
| 761 | KANSL1 |
| 762 | NEAT1 |
| 763 | SMARCA4 |
| 764 | LOX |
| 765 | CYBB |
| 766 | KCNJ2 |
| 767 | RAF1 |
